# Left Ventricular Noncompaction Is Associated with Valvular Regurgitation and a Variety of Arrhythmias

**DOI:** 10.3390/jcdd9020049

**Published:** 2022-02-02

**Authors:** Qing Li, Lianjie Miao, Lihong Xia, Hala Y. Abdelnasser, Fang Zhang, Yangyang Lu, Anika Nusrat, Mantasha Tabassum, Juxiang Li, Mingfu Wu

**Affiliations:** 1 Department of Cardiovascular Medicine, The Second Affiliated Hospital of Nanchang University, Nanchang 330006, China; liqing199137@126.com (Q.L.); xlh016@126.com (L.X.); zhangfang2056@163.com (F.Z.); 2 Department of Molecular and Cellular Physiology, Albany Medical College, Albany, NY 12208, USA; lmiao4@central.uh.edu (L.M.); ylu37@central.uh.edu (Y.L.); 3 Department of Pharmacological and Pharmaceutical Sciences, College of Pharmacy, University of Houston, Houston, TX 77204, USA; habdelna@CougarNet.UH.EDU (H.Y.A.); anusrat2@CougarNet.UH.EDU (A.N.); mtabassu@CougarNet.UH.EDU (M.T.)

**Keywords:** trabeculation, left ventricular noncompaction, right ventricular noncompaction, arrythmia, regurgitation, ventricular contraction

## Abstract

Left ventricular noncompaction (LVNC) is a type of cardiomyopathy characterized anatomically by prominent ventricular trabeculation and deep intertrabecular recesses. The mortality associated with LVNC ranges from 5% to 47%. The etiology of LVNC is yet to be fully understood, although decades have passed since its recognition as a clinical entity globally. Furthermore, critical questions, i.e., whether LVNC represents an acquired pathology or has a congenital origin and whether the reduced contractile function in LVNC patients is a cause or consequence of noncompaction, remain to be addressed. In this study, to answer some of these questions, we analyzed the clinical features of LVNC patients. Out of 9582 subjects screened for abnormal cardiac functions, 45 exhibit the characteristics of LVNC, and 1 presents right ventricular noncompaction (RVNC). We found that 40 patients show valvular regurgitation, 39 manifest reduced systolic contractions, and 46 out of the 46 present different forms of arrhythmias that are not restricted to be caused by the noncompact myocardium. This retrospective examination of LVNC patients reveals some novel findings: LVNC is associated with regurgitation in most patients and arrhythmias in all patients. The thickness ratio of the trabecular layer to compact layer negatively correlates with fractional shortening, and reduced contractility might result from LVNC. This study adds evidence to support a congenital origin of LVNC that might benefit the diagnosis and subsequent characterization of LVNC patients.

## 1. Introduction

Left ventricular noncompaction (LVNC: OMIM No. 604169) is a type of cardiomyopathy anatomically characterized by prominent ventricular trabeculation and deep intertrabecular recesses [1,2,3]. It was reported for the first time in 1969, being addressed as a spongy myocardial condition back then [4]. In the following years, LVNC has gained tremendous attention due to the improvements in cardiac imaging techniques, primarily echocardiography and magnetic resonance imaging, that have enabled more detailed visualization and increased clinical awareness of this syndrome. Subsequently, many LVNC cardiomyopathy cases were reported, and AHA enlisted LVNC as a type of cardiomyopathy in 2006 [3]. LVNC shows highly variable clinical manifestations ranging from asymptomatic to symptomatic, and the major clinical features of LVNC are heart failure, arrhythmias, thromboembolic events, and sudden death [5]. Its symptoms are progressive, considered the 3rd most common cardiomyopathy in the pediatric population, and the mortality of patients with LVNC ranges from 5% to 47% [6,7,8]. Despite its clinical significance, the mechanism of trabecular compaction and the etiology of LVNC are unknown. Moreover, whether LVNC is acquired or congenital cardiomyopathy has been an unraveled controversy [9]. The focus of most clinical investigations on isolated adult LVNC patients failed to trace the anomalies in embryonic developmental stages, and the knowledge gap regarding the molecular level regulation of trabeculation could be the possible reason [10,11].

As abnormalities in trabecular and ventricular morphogenesis lead to LVNC, the revelation of the biological and physiological development of trabecular formation and ventricular compaction will help elucidate the etiology of LVNC [12,13]. Trabeculae are sheet-like structures extending from the myocardium to the heart lumen and function to increase surface area to support nutrition and oxygen supply when the coronary system is not yet established [14]. Many studies show that a lack of trabeculation causes embryonic demise, and excess trabeculation causes LVNC [2,6,11,13,15,16,17,18,19,20,21,22,23,24,25,26,27,28,29,30,31,32,33,34,35]. The cellular and molecular mechanisms of trabecular formation have been revealed over the years. Recent studies demonstrated that the polarity-dependent oriented cell division (OCD) and directional migration of cardiomyocytes in the single-cell-thick myocardium contribute to trabecular initiation, resulting in myocardium with multiple-layer cells in mice [13,36,37,38,39]. However, in zebrafish, trabeculae are formed by polarity-dependent directional migration only [33,34,40]. Further study found that the cardiomyocyte in the outer and inner layers of the compact zone display different orientations during trabecular initiation, and disruption of their cellular orientation will result in trabecular initiation defects and LVNC [13,41]. Moreover, perpendicular OCD has contributed to trabecular specification and is responsible for trabecular cardiomyocytes being distinct from the cardiomyocytes in the compact zone [36]. After the trabecular initiation, the myocardium contains multiple layers of cardiomyocytes, and endocardial cells can burrow into the loose cardiomyocytes and separate them to form trabeculae [42,43].

Compared to the processes governing trabecular formation, the mechanism of ventricular compaction is mainly unknown. Recent studies report that endothelial initiated angiogenesis and Semaphorin 3E/PlexinD1 signaling might be required for ventricular compaction [44,45]. Another study along these lines shows that the trabecular cells coalesce with the compact zone to thicken and strengthen the compact zone [46]. However, the genetic networks and a clear description of the compaction process are not established yet. Furthermore, significant questions, i.e., whether the cause of LVNC is over-trabeculation or compaction arrest [12], whether LVNC represents an acquired pathology or has a congenital origin, whether LVNC patients associate with other clinical features, and whether the reduced contractile function is a cause or consequence of LVNC, are unknown. A thorough analysis of the clinical features of the LVNC will help answer these questions and identify the etiology of LVNC.

To answer some of these questions, we carefully examined the clinical features of LVNC patients. In this study, we explored a database of 9582 subjects screened for abnormal cardiac functions and found that 46 patients displayed the features of noncompaction. The hearts’ deformations were evaluated from the images harvested by echocardiography (ECHO) and/or cardiovascular magnetic resonance imaging (MRI). While echocardiography can be used as the first tracing tool in LVNC diagnosis, MRI-mediated evaluation can determine subclinical alteration in myocardial function and is more responsive to detect subtle functional changes and heart structures than ECHO [47].

The data show that 42 of the 46 patients display different extents of valvular or atrioventricular regurgitation. A total of 39 of the 46 patients present reduced systolic contraction, and 46 out of the 46 patients manifest different formats of arrhythmias that are not restricted to the noncompact myocardium. Interestingly, 1 out of the 46 patients manifests a right ventricular noncompaction (RVNC) but not a contractile defect, and 1 displays both LVNC and RVNC. This close retrospective examination of LVNC patients reveals some novel findings, including that LVNC is associated with regurgitation and arrhythmias in most of the patients and that the reduced contractility in LVNC patients correlates with the age of the patients and might be a consequence of LVNC. These findings suggest that regurgitation and arrhythmia might be clinical features for LVNC and add evidence to favor the notion that LVNC has a congenital origin.

## 2. Materials and Methods

### 2.1. Study Population and Clinical Data

The echocardiogram database, which includes 9582 unrelated patients screened for heart-related diseases at Second Affiliated Hospital of Nanchang University between November 2014 and November 2021, was searched for patients with the diagnosis of noncompaction. A total of 46 patients who display noncompaction were included in the analysis. Diagnosis of noncompaction was based on a consensus of re-evaluated echocardiography and MRI, according to the Jenni and Chin criteria and a dedicated participating cardiologist [15,48]. Clinical data were retrieved retrospectively from the medical records, including age, sex, cardiac diagnosis, electrocardiography, echocardiography, and cardiac MRI when available. The clinical symptoms, primary diagnosis, New York Heart Association classification (NYHA), associated dysmorphic features, valvular regurgitation, congenital heart disease, and presence of arrhythmia were documented. Echocardiograms were analyzed for ejection fraction, fractional shortening, and ventricular dimensions. Myocardial thickness was also determined at the site of the most prominent trabecular meshwork. The study design was approved by the Ethics Committee of the Nanchang University, Nanchang, China. All data used for this study were handled anonymously.

### 2.2. Diagnosis of LVNC and RVNC

Diagnosis of LVNC or RVNC is made using transthoracic echocardiography as a main diagnostic tool. Ventricular noncompaction is described by the compacted thin epicardial layer and a thicker noncompacted endocardial layer with a ratio between noncompacted to compacted myocardium >2 in end-systole of a short-axis slice [15,48]. Clinical data of cardiovascular MRI is frequently used to confirm or rule out the diagnosis.

### 2.3. Ventricular Systolic Funciton

Left ventricular (LV) systolic dysfunction was defined as LV ejection fraction of <50% or fractional shortening of <25% in both men and women on echocardiography.

### 2.4. Vavular Regurditation

Valvular regurgitation severity is determined by the regurgitant fraction (RF) according to the published reference [49], and if the RF is less than 30%, it is defined as mild; if RF is between 30–39, it is defined as moderate; and if the RF is larger than 40, it is defined as severe.

### 2.5. Statistics

The differences in contractility among the groups of three different T/C ratios and between two age groups are compared via non-parametric tests for statistical comparison. A *p*-value of 0.05 or less was considered statistically significant.

### 2.6. Statement

All methods were carried out in accordance with relevant guidelines and regulations.

## 3. Results, Figures and Tables

### 3.1. Clinical Symptoms of LVNC Patients

**Figure 1:**
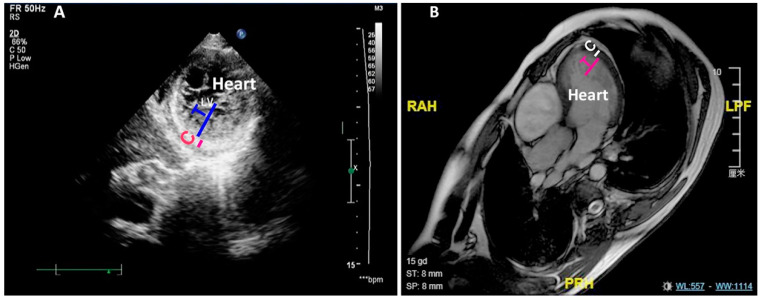
LVNC is detected by ECHO and MRI. (**A**) Shows the noncompaction measured by ECHO of an LVNC patient. (**B**) Shows the noncompaction measured by MRI of an LVNC patient. C: compact zone; T: trabecular zone; LV: left ventricle; ECHO: echocardiography; MRI: magnetic resonance imaging.

The characterization of LVNC is evolving but remains incomplete, as many questions remain to be settled. One of them is whether LVNC patients associate with novel clinical features in addition to the reported symptoms. We searched the database for cardiac noncompaction from 9582 echocardiograms performed on subjects who were admitted to the hospital for further clinical examination and potential heart-related diseases. The abnormal compaction was identified based on Chin and Jenni criteria with a ratio of NC/C larger than two (Figure 1A,B, and Table 1) [15,48].

Using these criteria, we identified 46 noncompaction cases. These patients account for 0.48% of all the cases searched in the database. Noncompaction occurs in different regions of the heart, primarily prominent in the apex of the left ventricle. Of the 46 patients, 44 display LVNC, 1 displays both LVNC and RVNC, and 1 displays RVNC. The age of the 46 patients ranges from 12 to 81 years old, and the average age is 52.2. Of the 46 patients, 32 were male, and 14 were female; 2 were younger than 15, and the other 44 patients were older than 20. The NYHA standard protocol was used to measure the severity of LVNC patients—24 patients showed grade I-II and 22 patients grade III-IV. Valvular regurgitation (40/46), reduced contractility (39/46), and arrhythmias (46/46) are the most common manifestations encountered in these patients. In this study, mild tricuspid and pulmonary regurgitation are not pathological, and they were not considered valvular regurgitation. Valvular Ebstein’s anomaly and atrial septal defects were also observed in some of the patients (Table 1). Unlike the previous report, about 80% of LVNC patients display neuromuscular disease [50]; only 1 out of 46 patients is associated with this defect. Cardiac functions, ventricular remodeling, and arrhythmias were examined and compared among patients (Table 1). We further characterize the features of the atrioventricular regurgitation, arrhythmias, reduced contractility, and right ventricular noncompaction of the 46 patients in the following sessions.

### 3.2. LVNC Patients Associate with Valvular Regurgitation

**Figure 2:**
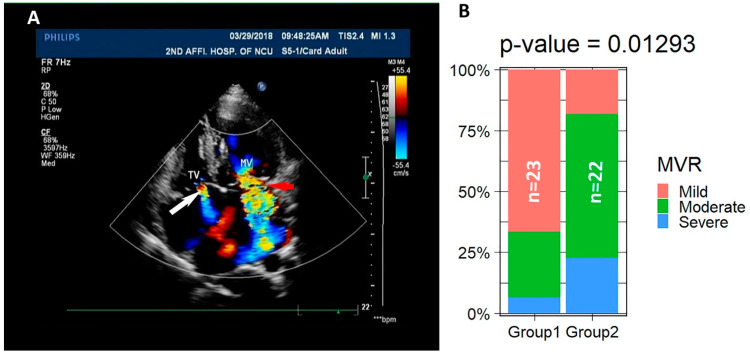
A total of 40 of the 45 LVNC patients are associated with valvular regurgitation. (**A**) ECHO images show the TVR indicated by the white arrow and MVR indicated by the red arrow. (**B**) The percentages of mild, moderate, and severe regurgitation in the two age groups are significantly different.

The valvular regurgitation in LVNC patients did not draw much attention until recently [6,51,52,53]. We examined the mitral valvular regurgitation (MVR), tricuspid valvular regurgitation (TVR), aortic regurgitation (AR), and pulmonary arterial regurgitation (PR) in all the patients based on the images/videos that were harvested via ECHO and/or MRI. Surprisingly, we found that 36 out of the 46 patients display MVR (Figure 2A and Table 1 and Table 2). A total of 36 out of 46 present TVR. Since mild TVR is not pathogenic, only the eight moderate or severe TVR are included to study the association of LVNC and valvular regurgitation (Figure 2A and Table 1 and Table 2). A total of 19 out of 46 have a clinical manifestation of AR (Table 1 and Table 2). A total of 6 out of the 46 display mild or moderate PR. Excluding the mild TVR and VR, we found that 40 patients showed clinical valvular regurgitation.

One of the major causes of regurgitation is the abnormal structure of the valves. We found that only #14 displays Ebstein’s anomaly with anterior longer and bigger leaflet, septal leaflet prolapse, and posterior leaflet deformity. The structure and components of the tricuspid and mitral valves of the other patients were not visibly abnormal. To determine if the symptoms are age-dependent, patients were divided into two groups: 23 patients in group 1 with age younger than the median age at 59 and 22 patients in group 2 with age equal to or older than 59. We analyzed the severity of MVR between the two age groups. It was well accepted that the prevalence of valvular heart diseases increases by age [54], and the severity of regurgitation between the two groups is significantly different based on Fisher’s Exact Test of the percentages of mild, moderate, and severe patients in the two groups (Table 2) (Figure 2B), suggesting that regurgitation is an age-related remodeling of the valves in the LVNC patients. These data imply that valvular regurgitation is associated with the onset of LVNC and might be a clinical feature for LVNC during the diagnosis.

### 3.3. The LVNC Patients Display Reduced Contractility and Thickness Ratio of Trabecular Layer to Compact Layer Negatively Correlates with Cardiac Contractility

**Figure 3:**
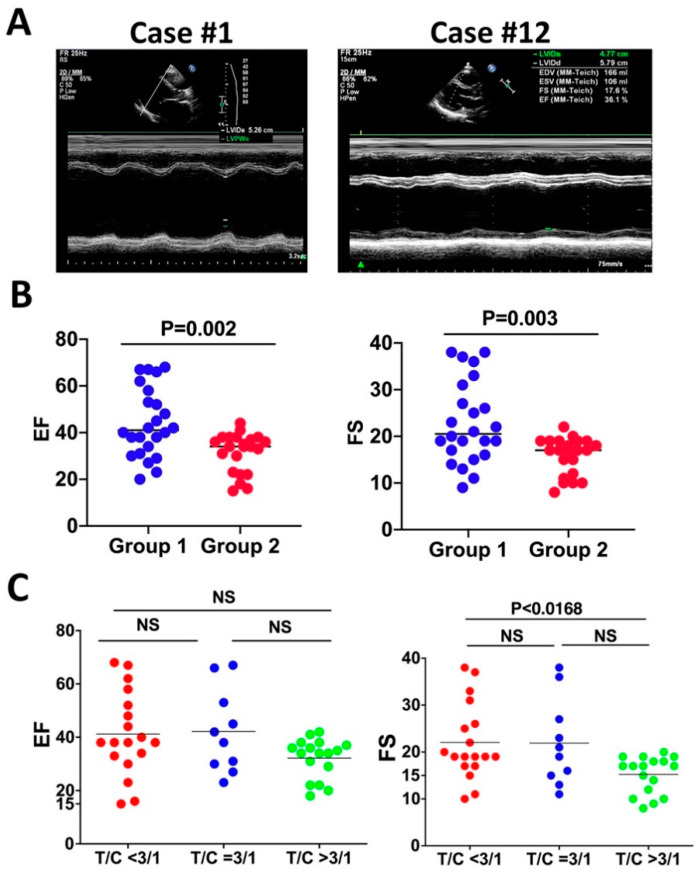
The LVNC patients display reduced contractility and thickness ratio of trabecular layer to compact layer negatively correlates with cardiac contractility. (**A**) Representative M-mode of left ventricular long-axis views of echocardiography. Case #1 displays reduced contractility, and Case #12 manifests normal contractility. (**B**) The ejection fraction (EF) and fractional shortening (FS) of group #1 (age younger than 59) are greater than group #2 (age older than 59), and the difference is not significant. (**C**) FS of patients with a T/C ratio higher than 3/1 is significantly less than patients with a T/C ratio smaller than 3/1.

Accumulating pieces of evidence indicate a crucial role for cardiac contraction and the resulting hemodynamic force on ventricular wall compaction [52,55]. Impaired contractile function and sluggish blood flow likely cause thrombotic formation in deep intertrabecular recesses of the left ventricle, which might underlie the frequent thrombotic events in LVNC. A prior study found that about 82% of the genetic mutations that caused LVNC occurred in genes that encode sarcomere components. Among them, MYH7, MYBPC3, and TTN are the most frequently mutated genes, and their mutations account for 71% of the LVNC cases [52]. Therefore, it has been speculated that the contractile function is required for trabecular compaction [56]. We examined the clinical data to determine the association between contractile dysfunction and LVNC. A total of 39 out of the 45 LVNC patients manifest reduced contractility as indicated by the reduced F.S. and E.F. (Table 1), suggesting that contractile dysfunction might be a feature of LVNC but is not necessarily the onset of LVNC. We compared the contractile functions between the two age groups to determine if reduced contractility correlates with age in LVNC patients. Compared to the normal range, 17 out of 23 patients in the young group show a contractile defect, indicated by reduced E.F. and F.S. (Figure 3A,B and Table 1), while 22 out of 22 patients in the old-age group display reduced contractility (Figure 3A,B and Table 1). Patients in the old-age group exhibited weaker contractility with a reduced ejection fraction (EF) and fractional shortening (FS), while the patients younger than 59 have a higher contractile function, but the difference is not significant based on the Mann–Whitney non-parametric test (Figure 3A,B). We further examined if the thickness ratio of trabecular layer to compact layer is correlated with the reduced systolic function. The 45 patients were divided into three groups: T/C < 3/1, T/C = 3/1, and T/C > 3/1. We compared the contractility of the three groups via the Kruskal–Wallis rank sum test and pairwise comparisons using Wilcoxon rank sum test. It was found that FS is significantly different, suggesting that a larger thickness ratio negatively correlates with the left ventricular systolic function (Figure 3C).

### 3.4. All LVNC Patients Are Associated with Arrhythmias

A key factor responsible for sudden death in LVNC patients is arrhythmia, a significant feature of LVNC. Ventricular arrhythmias are the standard prominent clinical components of LVNC [57]. It was thought that the deep intramyocardial invagination, which carries the Purkinje system deeper into the myocardium, might result in delayed depolarization and inhomogeneous repolarization and subsequently cause arrhythmias in LVNC [58].

**Figure 4:**
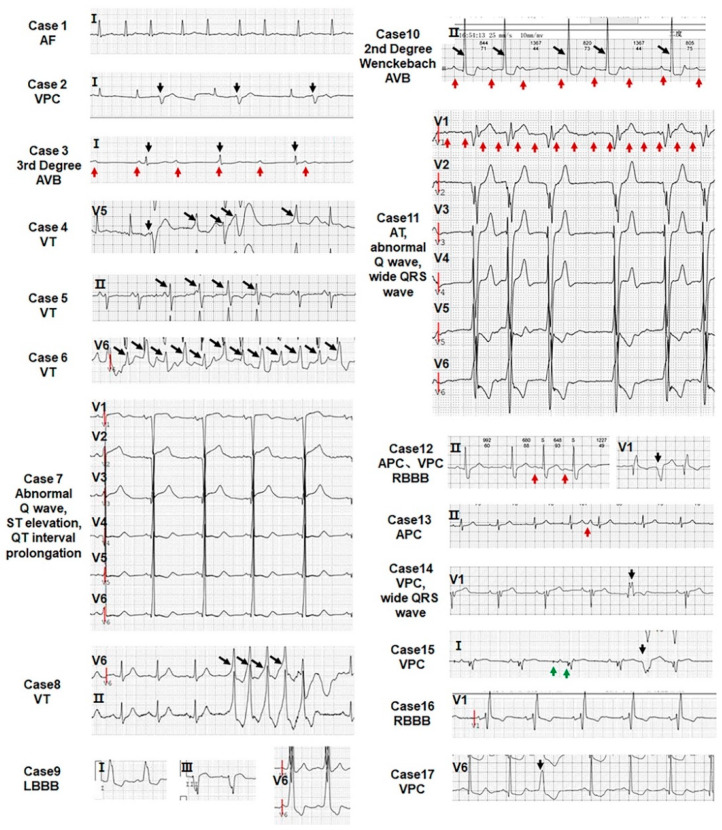
All patients show abnormal ECG. The type of arrhythmia of 17 LVNC patients is indicated in each echocardiogram. The red arrow points to the atrial wave, the black arrow points to the ventricular wave, and the green arrow directs to the pacemaker signal.

However, arrhythmias in the 46 patients are not restricted to the noncompacted myocardium, as atrial fibrillation (AF) is observed in LVNC patients [58]. LVNC patients bear mutations in RYR2 [59] and KCNH2/KCNQ1 [60] associated with cardiac arrhythmia syndromes. The range of percentages of arrhythmias in LVNC patients varies from 26 to 94 in different studies [48,52,61,62,63,64,65,66,67]. The major types of arrhythmias include ventricular tachycardia (VT) and AF. In this study, all the patients, including LVNC and RVNC patients, display arrhythmias and, in addition to VT and AF, supraventricular tachycardia (SVT), left bundle branch block (LBBB), atrial tachycardia (AT), atrial premature contraction (APC), sick sinus syndrome (SSS), right bundle branch block (RBBB), ventricular premature contraction (VPC), atrial ventricular block (AVB), and malignant arrhythmia (MA) were observed (Table 1 and Figure 4). Therefore, our data suggest that arrhythmias in LVNC patients are not restricted to the noncompacted myocardium.

### 3.5. RVNC Does Not Associate with Reduced Contractility

**Figure 5:**
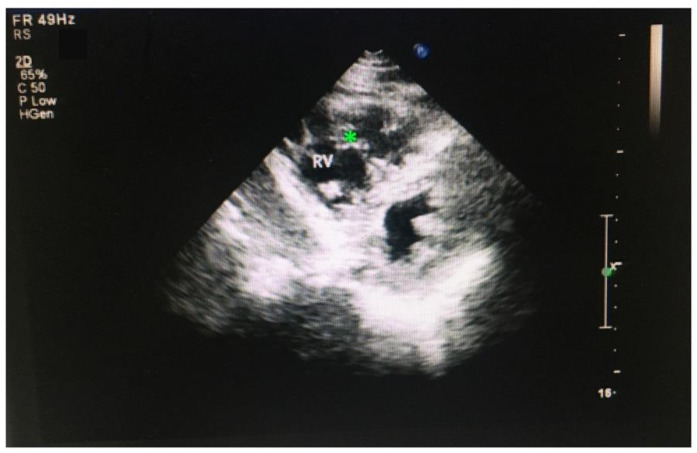
ECHO image of an RVNC. * indicates the trabecula in RV.

While the most common site of noncompaction is the left ventricle, the noncompaction in the right ventricle was rarely reported. It is likely that noncompaction in the left ventricle has more prominent clinical symptoms than noncompaction in the right ventricle which rendered noncompaction in the right ventricle to receive less attention being explored. Another possibility is that the rate of noncompaction in the right ventricle is low and rarely detected. In this study, of the 46 patients who display noncompaction, a 55-year-old male displays RVNC but not LVNC (Figure 5), and a 49-year-old male displays both RVNC and LVNC, which is considered as LVNC for the analysis.

Further examination revealed that RVNC is associated with dilated RV (>35 mm, normal range <25 mm), enlarged RA (>43, normal range 30–40 mm), and increased pulmonary artery pressure. The interventricular septum displays a normal thickness. The right ventricular wall is thinner. Trabeculae are prominent in the right ventricle and the apex of the right ventricle displays a beehive structure due to the abundance of trabeculae (Figure 5). The patient also displays atrial premature contraction (APC) (Figure 4) and regurgitation in the tricuspid valve (Table 1 and data not shown). However, left ventricle remodeling and cardiac functions are not affected (Table 1). The systolic and diastolic functions of the left ventricle are in the normal ranges (Table 1 and data not shown), suggesting that this patient does not display a reduced contractile defect in the left ventricle, which happens to be consistent with a previous study reporting that a RVNC patient displays normal contractile function in the RV and LV [68]. The patient has been smoking and drinking for more than 30 years. The patient denied further MRI examination.

## 4. Discussion

With the data reported in this study and data published by other groups, we would like to discuss further whether LVNC has congenital or acquired originations and whether the reduced contractility is a cause or a consequence of LVNC.

### 4.1. Congenital LVNC or Acquired LVNC

LVNC is characterized by hypertrabeculation and noncompaction in myocardial anatomy associated with deep intertrabecular recesses [1,2,3,69]. Clinical presentation of LVNC is progressive and heterogeneous, and some symptoms are not recognized and accepted, which prevents setting a universal standard to characterize LVNC and to determine the etiology of LVNC. A significant block for elucidating the etiology of LVNC is whether it represents an acquired pathology or has a congenital origin. Understanding the trabecular formation and subsequent compaction will help identify the origins and etiologies of LVNC. There are two major steps in trabecular formation. The first step is the polarity-dependent OCD, cell orientation, and directional migration of cardiomyocytes in the early myocardium, which contribute to trabecular initiation, resulting in myocardium with multiple-layer cells in mice [13,36,37,38,39,41]. Subsequently, the endocardial cells burrow into the multiple-layer myocardium and separate cardiomyocytes to form trabeculae [42,43]. After trabecular formation, the myocardium will undergo compaction through gradually compacting inwards from the base to the apex [61,70], and the trabeculae will coalesce with the compact zone to form the thickened compact zone [46]. Therefore, the etiology of LVNC can be based either on over-trabeculation or compaction arrestment. Both over-trabeculation and compaction arrest occur during the cardiac developmental stage and are thereby considered to bear a congenital origin [12]. Several pediatric studies indisputably demonstrated that LVNC possesses a congenital origin in children [1,20]. However, it was also found that LVNC can be acquired as many LVNC patients did not display noncompaction defects until a later stage, suggesting an acquired origin of LVNC. Therefore, it is likely that there are two types of LVNC: congenital LVNC and acquired LVNC.

Nevertheless, we would provide a different opinion: LVNC is congenital, but the clinical symptoms can be manifested in embryos/childhood or adulthood depending on the underlying genetic mutations. Although it was shown that coronary angiogenesis [44], Semaphorin 3E/PlexinD1 [45], Notch1 signaling [71], and many genes, including Numb [13] and DAAM1 [72], are involved in ventricular compaction, the genetic networks that regulate trabecular compaction are still not established yet, and a unifying description of the compaction process is not available. However, it is agreed that the ventricular compaction is a complicated process and regulated by complex signaling networks, and different gene mutations present different phenotypes. Some genes are required for trabecular compaction and embryonic development, and the mutated animals bearing mutations of these genes will display LVNC at an early postnatal stage or even die during pregnancy. While some genes regulate the maintenance of compaction and cardiac homeostasis, their mutations will cause LVNC at a later stage. Therefore, we infer that LVNC is congenital, and the clinical symptoms we observed in human subjects are manifestations of abnormal/mutated gene function at different stages.

The two different manifestations of LVNC can be modeled in the mouse. LVNC, with the congenital onset, usually will cause embryonic lethality or early neonatal death in the mouse. The adult-onset of LVNC was not modeled in the mouse until a recent report that compound heterozygous mutant mice provide an adult-onset mouse model [73]. This study shows that the adult-onset LVNC mouse model can be established unless the gene-based dosage is not lethal so that the animals can survive to the adult stage and display LVNC. In summary, we propose that LVNC is a congenital defect, and the manifestation can be congenital and adult onsets depending on the functions of the disrupted genes.

In this study, our results support the notion that LVNC is congenital, and the clinical symptoms are manifested at congenital or adult stages. With a detailed clinical diagnosis of the 46 noncompaction patients, we found some novel features of LVNC; most of the noncompaction patients are associated with regurgitation, and all the patients are associated with arrhythmias. The LVNC was supposed to be associated with ventricular related arrhythmias such as VPC and VT but not atrial related arrhythmias such as AT, APC, SSS, AVB, and AF. Some of the arrhythmias might be acquired due to ventricular remodeling. However, this could only explain the acquisition of ventricular arrhythmias but not the etiology of atrial related arrhythmias. A possible explanation is that some of the arrhythmias result from cardiac remodeling, but even this could not explain the etiology of SSS, VPC, AVB, and other types of arrhythmias observed in this study. Instead, these data suggest a congenital formation of these arrhythmias that associate with LVNC. In summary, manifestations of different types of arrhythmias suggest that the arrhythmias, or at least some of the arrhythmias in LVNC patients, are congenital.

One of the major causes of regurgitation is the valvular structural defect. Of the 45 LVNC patients, only #14 displays Ebstein’s anomaly. The structure and components of the tricuspid and mitral valves of the other patients were not noticed to be abnormal. However, many components of the valve, including the annulus, leaflets, chords, papillary muscles, and ventricular function and geometry can contribute to regurgitation. A more detailed examination of the valves in the LVNC patients will be needed to determine if the regurgitation is caused by congenital structural defects or remodeled structural changes. An alternative way to determine if the regurgitation symptoms are acquired by remodeling or are congenital is to determine whether the regurgitation symptoms are being associated with age or not. We analyzed the severity of regurgitation between the two age groups. It was well accepted that the prevalence of valvular heart diseases increases by age [54], and the severity of regurgitation between the two groups is significantly different based on the percentage of mild, moderate, and severe patients in the two groups (Table 2), suggesting that regurgitation is an age-related remodeling of the valves in the LVNC patients. These data imply that valvular regurgitation is associated with the onset of LVNC and might be a clinical feature for LVNC during diagnosis.

### 4.2. Reduced Contractility Is a Cause or a Consequence of LVNC

Accumulating pieces of evidence indicate a crucial role of cardiac contraction and the resulting hemodynamic force on ventricular wall compaction [52,55]. Mutations of many genes, including MYH7, MYBPC3, and TTN, that encode sarcomere components cause LVNC [52]. Of the genes whose mutations would affect contraction and develop LVNC, MYH7 was the only sarcomere gene associated with CHD [52]. Therefore, it has been speculated that the contractile function is required for trabecular compaction [56]. The impaired contractile function causes sluggish blood flow, likely resulting in thrombotic formation in deep intertrabecular recesses of the left ventricle, which might be the reason for frequent thrombotic events in LVNC. However, whether the reduced contractile function in LVNC is a cause or consequence is unknown.

In this study, we examined the clinical data and found that 39 out of the 45 LVNC patients display reduced contractility, suggesting that contractile function might be a feature of LVNC. However, 6 out of 45 LVNC patients did not display reduced contractility, suggesting that reduced contractility is not required for the onset of LVNC. We compared the contractile functions between the two age groups and found that 16 out of 23 patients in the young group display a contractile defect, while 22 out of 22 patients in the other group display reduced contractility (Table 1 and Figure 3). The differential contractile defect between the two groups suggests that the onset of the reduced contractility could be an acquired feature in LVNC. A previous study quantified trabeculations in a large cohort of otherwise healthy adults by MRI, which determined the associations of trabeculae with cardiac function and concluded that greater trabeculations are associated with decreased LV function [74]. Consistently, our studies found that the thickness ratio of trabecular layer to compact layer negatively correlates with reduced systolic function. These data suggest that reduced contractility might be a consequence of LVNC and is not necessarily required for the onset of LVNC.

However, this study has some limitations as following monitoring of the contractility changes in LVNC patients in a time-dependent manner will be needed to make a decisive conclusion regarding whether contractility is a cause or consequence of LVNC. Furthermore, due to the small sample size, the limited cases of LVNC do not reflect the etiology of all the LVNC patients and do not include all the causes that onset the LVNC in patients. This study used the ECHO, which is less sensitive than cardiac MRI, to screen LVNC patients, which might be why only 0.48% of subjects display LVNC.

## 5. Conclusions

In summary, we analyzed the clinical symptoms of 45 LVNC and 1 RVNC patient from 9582 echocardiograms performed on subjects with potential heart diseases. We found that 40 out of 46 LVNC patients showed clinical valvular regurgitation, 39 of the 45 LVNC patients display reduced systolic contraction, and 46 out of the 46 patients display various forms of arrhythmia. This retrospective analysis reveals novel findings that LVNC is associated with regurgitation in most of the patients and arrhythmias in all the patients, aged LVNC patients have a tendency of reduced contractility than young LVNC patients, and thickness ratio of trabecular to compact negatively correlates with the reduced systolic function. The findings suggest that regurgitation might be a clinical feature for LVNC. LVNC is a congenital defect, and the manifestations can be present in the congenital or adult stage depending on the functions of the disrupted genes.

## Figures and Tables

**Table 1:** Clinical features of the 45 LVNC and 1 RVNC patient.

Case #	Sex	Age	Arrhythmia	LVNC	T/C	EF (50–70%)	FS (>25%)	NYHA	LVd (35–55)	LVs (25–37)
1	M	39	AF	Y	2/1	48	25	II or III	70	53
2	M	68	VPC	Y	5/1	38	19	I	52	42
3	F	27	III°AVB	Y	3/1	66	36	I	51	33
4	F	12	VT	Y	3/1	67	38	I	54	33
5	M	62	VPC, VT	Y	4/1	22	10	II or III	58	52
6	M	14	VT	Y	3/1	30	15	IV	64	55
7	F	81	AQW, STE, QTIP	Y	5/1	41	20	III	56	45
8	M	29	VT, VPC	Y	3/1	53	27	I	48	35
9	F	60	LBBB	Y	2/1	38	19	II or III	66	54
10	M	35	Ⅱ°Ⅰ AVB	Y	2/1	62	33	II	42	28
11	M	41	AT, AQW	Y	3/1	31	16	III	58	49
12	M	71	VPC, APC, RBBB	Y	4/1	36	18	III	58	48
13	M	55	APC	RVNC	2.5/1	68	38	I	47	29
14	M	31	VPC, wide QRS wave	Y	2.5/1	52	26	II	41	30
15	M	28	SSS, VPC	Y	3/1	23	11	III	64	56
16	M	59	RBBB	Y	5/1	18	8	II	60	55
17	M	57	VPC	Y	2.5/1	58	31	II	48	33
18	M	40	SB, STE, TWC	Y	3/1	42	21	II or III	58	45
19	M	61	AF, VPC, RBBB	Y	3.5/1	35	17	IV	63	52
20	F	75	AT, VPC, VT	Y	2.5/1	44	22	II or III	59	45
21	M	62	AF, VPC, STE, ST-TWC	Y	3.5/1	22	10	IV	91	81
22	M	49	SB, AQW	RVNC LVNC	>3/1	40	22	I	47	37
23	M	65	ST, LBBB, ST-TWC	Y	3/1	38	19	II or III	71	59
24	M	70	AQW, ST-TWC	Y	5/1	34	17	II or III	72	60
25	F	71	ST-TWC,	Y	2.5/1	30	15	II or III	65	56
26	M	63	VPC, AQW	Y	5.6/1	38	12	IV	72	58
27	M	42	ST-TWC	Y	2.5/1	67	37	I	46	29
28	M	55	VPC	Y	3/1	45	23	II	60	46
29	F	22	ST-TWC, Q-TIP	Y	3/1	27	13	II	68	59
30	F	42	Atrial flutter, VPC	Y	2.5/1	38	19	II	62	50
31	M	61	LBBB	Y	2.3/1	16	17	II or III	86	79
32	M	32	AQW	Y	>3/1	34	17	II	66	55
33	M	78	VPC, VT	Y	>3/1	36	18	II	68	56
34	M	68	AF	Y	3.6/1	34	17	II or III	65	54
36	M	37	APC	Y	4.4/1	42	19	II	66	54
37	M	21	ST, AQW, ST-TWC	Y	>3/1	29	14	IV	64	55
38	F	72	LBBB	Y	2.4/1	23	11	II	64	57
39	F	68	VPC, LBBB	Y	2.6/1	15	10	III	59	53
40	M	66	AF, AVB, LBBB, VPC	Y	2.6/1	33	19	III	82	67
41	F	48	AF, ST-TWC	Y	2.6/1	40	20	II	55	44
42	M	58	AF, LBBB	Y	5.2/1	20	9	II	79	71
43	F	57	VPC	Y	2.4/1	38	19	II or III	63	52
44	M	60	AQW	Y	5.0/1	31	15	II	65	55
45	M	57	AF	Y	>2/1	38	19	II	70	57
46	F	67	LBBB	Y	3.8/1	37	19	II	61	49
47	F	63	P-RIP, TWC	Y	2.5/1	34	17	II	70	59

AQW: abnormal Q wave; STE: ST elevation; QTIP: QT interval prolongation; VT: ventricular tachycardia; AF: atrial fibrillation; SVT: supraventricular tachycardia; LBBB: left bundle branch block; AT: atrial tachycardia; APC: atrial premature contraction; SSS: sick sinus syndrome; RBBB: right bundle branch block; VPC: ventricular premature contraction; AVB: atrial ventricular block; TWC: T wave change; RIP: R interval prolongation; MA: malignant arrhythmia; RVNC: right ventricular noncompaction. The number highlighted in red indicates an out of normal range.

**Table 2:** A total of 40 of the 45 LVNC patients display valvular regurgitation.

Case #	Gender	Age	Valvular Regurgitation
1	M	39	Severe MVR, severe TVR, Moderate PR, Mild AR
2	M	68	Mild MVR, mild TVR, mild AR
3	F	27	Mild MVR, mild TVR, mild AR
4	F	12	Moderate MVR, mild TVR, mild AR
5	M	62	Severe MVR, mild TVR
6	M	14	Moderate MVR, mild TVR
7	F	81	Moderate MVR, mild TVR, mild PR
8	M	29	Mild TVR
9	F	60	Moderate MVR, mild TVR, mild AR
10	M	35	Mild TVR
11	M	41	Moderate MVR, mild TVR, mild AR
12	M	71	Mild MVR, mild TVR
13	M	55	Moderate TVR, mild PR
14	M	31	Moderate TVR, Ebstein’s anomaly
15	M	28	Mild MVR, mild TVR, mild AR
16	M	59	Moderate MVR, mild TVR
17	M	57	Mild MVR, mild TVR
18	M	40	Mild MVR, mild TVR
19	M	61	Moderate-severe MVR, moderate-severeTVR, mild AR
20	F	75	Moderate MVR, mild-moderate TVR, mild AR
21	M	62	Moderate-severe MVR, mild TVR, mild AR
22	M	49	Mild-moderate TVR
23	M	65	Moderate MVR, mild-moderate TVR
24	M	70	MVR prolapse, mild AR
25	F	71	Mild-moderate MVR, Mild-moderate AR
26	M	63	Mild-moderate MVR
27	M	42	-
28	M	55	Mild MVR, mild TVR
29	F	22	-
30	F	42	Mild MVR
31	M	61	Mild MVR
32	M	32	Moderate MVR, mild TVR
33	M	78	Mild MVR, mild TVR, mild AR
34	M	68	Severe MVR, mild TVR, mild AR
36	M	37	-
37	M	21	Mild MVR, mild TVR
38	F	72	Moderate MVR, mild TVR, mild AR, mild PR
39	F	68	Moderate-severe MVR, mild-moderate TVR, mild AR, mild PR
40	M	66	Moderate MVR, mild TVR, mild AR
41	F	48	Mild MVR, mild TVR
42	M	58	Mild MVR, mild TVR
43	F	57	-
44	M	60	Moderate MVR, mild TVR
45	M	57	Mild-moderate AR, mild MVR, mild TVR, mild PR
46	F	67	Mild-moderate MVR, mild TVR, mild AR
47	F	63	Moderate MVR

MVR: mitral valvular regurgitation; TVR: tricuspid valvular regurgitation; AR: aortic regurgitation; PR: pulmonary arterial regurgitation. Since mild TVR and PR are not pathologic, they are not included in the association between LVNC and valvular regurgitation.

## Data Availability

All data supporting reported results in this paper are provided in the figures and tables in this paper.
